# Whole-Genome Analysis of Multidrug-Resistant *Salmonella* Enteritidis Strains Isolated from Poultry Sources in Korea

**DOI:** 10.3390/pathogens10121615

**Published:** 2021-12-10

**Authors:** Tae-Min La, Taesoo Kim, Hong-Jae Lee, Joong-Bok Lee, Seung-Yong Park, In-Soo Choi, Sang-Won Lee

**Affiliations:** College of Veterinary Medicine, Konkuk University, Seoul 05029, Korea; fkxoals@konkuk.ac.kr (T.-M.L.); taesoo958111@konkuk.ac.kr (T.K.); lhj90@konkuk.ac.kr (H.-J.L.); virus@konkuk.ac.kr (J.-B.L.); paseyo@konkuk.ac.kr (S.-Y.P.); ischoi@konkuk.ac.kr (I.-S.C.)

**Keywords:** whole-genome sequencing, *Salmonella* Enteritidis, multidrug resistance, virulence plasmid, salmonellosis

## Abstract

The *Salmonella* Enterica subsp. Enterica serovar Enteritidis is one of main serovars isolated from human patients with food poisoning and poultry without clinical signs. Consumption of poultry products contaminated with *Salmonella* Enteritidis is a common source of human salmonellosis; 82 *Salmonella* spp. were isolated from 291 samples of retail chicken meat, 201 one-day-old chicks, 30 internal organs of chickens, 156 chicken eggs, 100 duck eggs, 38 straw bedding samples, 18 samples of retail duck meat, and 19 swab samples from slaughterhouses in 2019 and 2020. An antibiotic susceptibility test was performed for all isolates, revealing 33 multidrug-resistant (MDR) strains. The whole genome of 33 MDR strains isolated in 2019 and 2020 and 10 strains isolated in 2011, 2012, and 2017 was sequenced using the MinION sequencing protocol. Within these 43 samples, 5 serovars were identified: *S.* Enteritidis, *S.* Agona, *S.* Virchow, *S.* Albany, and *S.* Bareilly. The most common serovar was *S.* Enteritidis (26/43), which showed the highest resistance to ampicillin (100%), followed by nalidixic acid (90%) and colistin (83%). Core genome multilocus sequence typing analysis showed that the *S.* Enteritidis strains isolated from different sources and in different years were clustered together. In addition, the *S.* Enteritidis strains isolated since 2011 consistently harbored the same antibiotic resistance patterns.

## 1. Introduction

Non-typhoidal *S.* Enterica causes foodborne salmonellosis and has become a global health threat [1]. *Salmonella* Enteritidis is frequently isolated from human patients with salmonellosis caused by the consumption of contaminated chicken meat and chicken products, such as eggs [1]. Fever, abdominal cramps, and diarrhea are common clinical symptoms of *Salmonella* Enteritidis infection in humans, which appear 12 to 72 h after consuming the contaminated food [2]. In most cases, the symptoms last 4 to 7 days and resolve on their own without the need for antibiotics. However, in the elderly, infants, and people with weakened immune systems, the diarrhea symptoms can be severe, and septicemia, and even death, can occur [3]. No clinical symptoms are observed in birds infected with *Salmonella* Enteritidis [4].

In Korea, antibiotic usage is not regulated in the poultry industry, to which about 1000 tons of antibiotics were sold between 2011 and 2017 [5]. In 2012, 720,000 tons of chicken meat were produced, which represents the second-largest protein source in Korea [6]. It is noteworthy that the prevalence of antibiotic-resistant *Salmonella* is high in retail chicken in Korea [5,7].

Recently, the isolation of multidrug-resistant (MDR) *Salmonella*, which is resistant not only to traditional first-line antibiotics such as ampicillin, chloramphenicol, and trimethoprim but also to currently recommended antibiotics such as fluoroquinolones and extended-spectrum cephalosporins, has increased dramatically [8,9]. MDR *Salmonella* with the same antibiotic resistance phenotype has been isolated from humans and poultry [10]. The spread of antibiotic resistance genes from animal bacteria to human populations is a major public health concern [11]. Investigation and epidemiological analysis of the MDR *Salmonella* in foods are necessary to prevent the development of the spread of MDR strains [12].

Epidemiological sources of *Salmonella* outbreaks have been investigated using pulsed-field gel electrophoresis (PFGE) and multiple-locus variable-number tandem repeats (MLVA) analysis [13]. In previous studies, these approaches have successfully detected the genetic relationship between *Salmonella* Enteritidis strains isolated from human patients or poultry sources in Korea [14,15]. However, the discrimination power of these methods for genetically closely related *Salmonella* Enteritidis strains is limited [16,17].

Whole-genome sequencing (WGS) has improved the resolution of genome analyses; thus, the sources of *Salmonella* outbreaks can now be traced [18,19,20]. Two analytic methods are commonly used for WGS-based genome analysis: single-nucleotide polymorphism (SNP) analysis and multilocus sequence typing (MLST) [21]. SNPs are identified by mapping sequence data of isolates to a reference genome and then recording the nucleotides that differ within the datasets [22]. The MLST method explores the allelic difference in a predefined set of gene loci [23]. In order to improve the resolution power of the MLST, the number of genes included in the scheme was increased [24]. Core genome MLST (cgMLST) balances the number of loci included in a scheme by considering those loci present in the majority of the isolates (ranging from 95% to 99%) in a given species [22,25].

Recently, several studies have used WGS to investigate the molecular relationship of *Salmonella* isolated from various sources [26,27,28,29]. However, only a small number of studies using WGS for the epidemiological analysis of *Salmonella* spp. have been reported in Korea [30,31,32].

Here, we have isolated 82 MDR *Salmonella* strains from 853 poultry sources in Korea and sequenced their genomes using the Oxford Nanopore approach. In order to investigate the relationships between the *Salmonella* Enteritidis strains, the whole genome sequences of the MDR isolates were compared using cgMLST and whole-genome SNP (wgSNP).

## 2. Materials and Methods

### 2.1. Sample Collection

Between 1 April 2019 to 11 May 2020, a total of 853 samples (291 samples of retail chicken meat, 201 one-day-old chicks, 30 internal organs of chickens for pet food, 156 chicken eggs, 100 duck eggs, 38 straw bedding samples, 18 samples of retail duck meat, and 19 swab samples from slaughterhouses) were collected. Retail chicken meats were purchased in two local supermarkets, three traditional markets, and from the internet. From each sample, 2–10 pieces of packed chicken meat were collected. Chicken eggs were purchased in one local supermarket. Retail duck meat samples were purchased in two local supermarkets and one traditional market. Duck eggs were purchased in one local supermarket and from online stores. Straw bedding samples from geographically separated multiple poultry farms were collected. Swab samples positive for *Salmonella*, as determined by polymerase chain reaction (PCR), from eight geographically separated slaughterhouses were collected. In addition, 11 *Salmonella* Enteritidis strains isolated in 2011, 2012, and 2017 were kindly provided by the Avian Disease Laboratory, College of Veterinary Medicine, Konkuk University, Korea.

### 2.2. Salmonella Isolation

For retail chicken, duck meat, and internal organs of chicken, each sample was aseptically placed in a sterile plastic bag containing 400 mL of buffered peptone water broth (BPW, Difco, Detroit, MI, USA) and shaken for 2 min. The rinsed material (20 mL) was vortex-mixed in 20 mL of BPW for 15 s, and then incubated at 37 °C for 24 h. For straw bedding and swab samples, each sample was aseptically placed in a sterile plastic bag containing 20 mL of BPW and shaken for 15 s. The rinsed material (0.1 mL) was incubated at 37 °C for 24 h. Chicken and duck egg samples were incubated in the egg incubator for 21 and 28 days, respectively. Liver, spleen, and cecal tonsil were collected from the egg embryo. The organs were placed into a sterile plastic bag containing 20 mL of BPW and homogenized using the stomacher for 2 min. The homogenized sample (0.1 mL) was vortex-mixed in 10 mL of Rappaport-Vassiliadis broth (RV, Difco, Detroit, MI, USA) and incubated at 37 °C for 24 h. Incubated BPW (100 µL) was vortex-mixed for 15 s in 10 mL of RV and then incubated at 41.5 °C for 20 h. The presence of *Salmonella* spp. in the incubated RV was analyzed by PCR, as described previously [33]. Samples that yielded positive results were streaked onto *Salmonella* ChromoSelect agar (Sigma-Aldrich, St. Louis, MO, USA), followed by incubation at 37 °C for 24 h. Pink colonies of *Salmonella* spp. on the agar were validated by PCR, and positive colonies were stored at −80 °C in glycerol.

### 2.3. Antibiotic Susceptibility Test

Antibiotic susceptibility was determined using the Sensititre panel (KRCDC2F; Thermo Fisher Scientific, Waltham, MA, USA) with the following antibiotics: ciprofloxacin (CIP, 0.03–0.5 µg), nalidixic acid (NAL, 2–128 µg), imipenem (IMI, 1–8 µg), colistin (COL, 2–16 µg), ampicillin (AMP, 2–64 µg), tetracycline (TET, 2–128 µg), chloramphenicol (CHL, 2–32 µg), azithromycin (AZI, 2–32 µg), gentamicin (GEN, 1–64 µg), streptomycin (STR, 2–128 µg), amikacin (AMI, 4–64 µg), trimethoprim/sulfamethoxazole (SXT, 1/19–16/304), cefotaxime (FOT, 1–32 µg), ceftriaxone (AXO, 1–32 µg), cefoxitin (FOX, 4–32 µg), and ceftazidime (TAZ, 1–16 µg), according to the Clinical and Laboratory Standards Institute guidelines (Wayne, PA, USA) [34]. Briefly, 10 µL portions of *Salmonella* spp. strains (1 × 10^5^ cfu/mL) cultured overnight were thoroughly mixed with 11 mL of Muller Hinton Broth with *N*-Tris (hydroxymethyl) methyl-2-aminoethanesulfonic acid; 50 µL portions were placed in the wells of the Sensititre panel. The panel was sealed with film, and the results were assessed manually after 24 h incubation at 37 °C. The minimum inhibitory concentration (MIC) was recorded as the lowest concentration of antibiotic that inhibited visible growth, identified as a turbidity or deposit of cells at the bottom of a wall. *Escherichia coli* (ATCC25922) was used as the quality control standard. *Salmonella* spp. resistant to more than three classes and more than one antibiotic in a single class were designated as an MDR strain.

### 2.4. Extraction and WGS of MDR Salmonella Genomic DNA

Genomic DNA was extracted from overnight cultured MDR *Salmonella* spp. using a MagAttract kit (Qiagen, Hilden, Germany) according to the manufacturer’s protocol. The purity and concentration of the extracted DNA were measured with a NanoDrop spectrophotometer (Thermo Fisher Scientific) and a Quantus fluorometer (Promega, Madison, WI, USA), respectively. A library was prepared for sequencing using native barcoding genomic DNA kits, and WGS sequencing was performed using the MinION system (Oxford Nanopore Technologies, Oxford, UK), as described by the respective manufacturers. The library was loaded onto FLO-MIN106 R9.4.1 flow cells and sequenced for 48 h. Data were base-called using Albacore (Oxford Nanopore Technologies). A library prepared using a TrueSeq Nano DNA instrument (Illumina, San Diego, CA, USA) was also sequenced using the HiSeq4000 system (Illumina, San Diego, CA, USA) for error correction of the nanopore sequencing results.

### 2.5. Assembly, Polishing, and Annotation of MDR Salmonella DNA

Reads generated from nanopore sequencing were downsampled to generate ~100× coverage depth of the *Salmonella* genome (4.9 Mb) using *seqtk* (https://github.com/lh3/seqtk; accessed on 22 September 2021). Downsampled reads were de novo assembled using the Flye algorithm [35] with default parameters. The assembled contigs were polished using unicycler_polish [36] with the Illumina fastq reads with default parameters. The assembled *Salmonella* genome was annotated using Prokka [37].

### 2.6. Data Analysis

MDR *Salmonella* serovar was predicted using SeqSero from assembled contigs [38]. CgMLST was determined using SeqSero. The minimum spanning tree of core genome MLST was visualized using GrapeTree [39]. Antibiotic resistance genes were identified using Resfinder [40]. SNPs between the whole genomes of the sequenced *Salmonella* Enteritidis strains in this study and those of the Korean *Salmonella* Enteritidis strains deposited in the public database were identified and aligned using kSNP3.0 [41] with the optimum kmer size 19. The genomic sequence of the *Salmonella* Enteritidis P125109 strain (GenBank no. NC011294) was used as the reference genome for SNP calling. A whole-genome SNP tree was constructed based on the pan SNPs generated by kSNP3.0 using RAxML, with the General Time Reversible gamma substitution model and 1000 bootstrap replicates. The phylogenetic tree with antibiotic resistance genes was visualized using the interactive Tree of Life version 5 (iTOLv5) (http://itol.embl.de/; accessed on 22 September 2021).

## 3. Results

### 3.1. Prevalence of Salmonella spp.

In total, 82 *Salmonella* spp. were isolated from 853 samples: 100% from swab samples from the slaughterhouses (19/19), 61% from retail duck meat (11/18), 26.7% from internal organs of chickens (8/30), 14.1% from retail chicken meat (41/291), 5.3% from straw bedding samples (2/38), and 0.5% from one-day-old chicks (1/201). No *Salmonella* spp. was detected from chicken and duck eggs.

### 3.2. Antibiotic Resistance Profiles of the Isolated Salmonella spp.

The antibiotic resistance profiles of the *Salmonella* spp. isolates are shown in Table 1. Among the 82 *Salmonella* spp. tested, 40% isolates were identified as MDR strains (33/82). All 10 *Salmonella* Enteritidis strains isolated in 2011, 2012, and 2017 were identified as MDR. The highest resistance rate was to ampicillin (100%, 43/43), followed by nalidixic acid (76.74%, 33/43), tetracycline (74.42, 32/43), and colistin (53.49%, 23/43). All MDR isolates were susceptible to imipenem, azithromycin, and amikacin.

### 3.3. Results of Whole-Genome Sequencing and In-Silico Serotyping of MDR Salmonella spp.

The genomic features of the MDR *Salmonella* spp. are shown in Table 2. The sequence data for all MDR isolates yielded a depth of greater than 100, except for one sample with a depth of 87.5. The genome assembly generated 1–5 contigs. The size of the chromosome was 4,547,043–4,878,409 bp. MDR isolates were assigned to five serovars. *Salmonella* Enteritidis was the most prevalent serovar (57.78%, 26/43) followed by *Salmonella* Agona (15.6%, 7/43), *Salmonella* Virchow (13.3%, 6/43), *Salmonella* Albany (8.89%, 4/43), and *Salmonella* Bareilly (2.2%, 1/43).

### 3.4. Antibiotic Resistance Profiles of MDR Salmonella Enteritidis

The highest resistance observed in MDR *Salmonella* Enteritidis strains was to ampicillin (100%, 26/26), followed by nalidixic acid (96.15%, 25/26), colistin (88.46%, 23/26), and tetracycline (69.23%, 18/26). Resistance to third-generation cephalosporins (cefotaxime, ceftriaxone, and ceftazidime) was observed in 16 isolates (46.15%). These isolates were also resistant to nalidixic acid, colistin, ampicillin, tetracycline, and gentamicin. Isolation sources of these isolates were internal organs of chicken, retail chicken meat, straw for bedding, and farm environment. MDR *Salmonella* Enteritidis strains that were resistant to colistin were also resistant to ampicillin and nalidixic acid. All the sequenced *Salmonella* Enteritidis strains harbored antibiotic resistance genes that coincided with antibiotic resistance phenotypes except colistin resistance (Table 3). All MDR *Salmonella* Enteritidis strains carried the aac(6)-Iaa gene. The mobile colistin resistance (*mcr*) gene and chromosomal mutations related to colistin resistance were not found.

### 3.5. CgMLST

**Figure 1:**
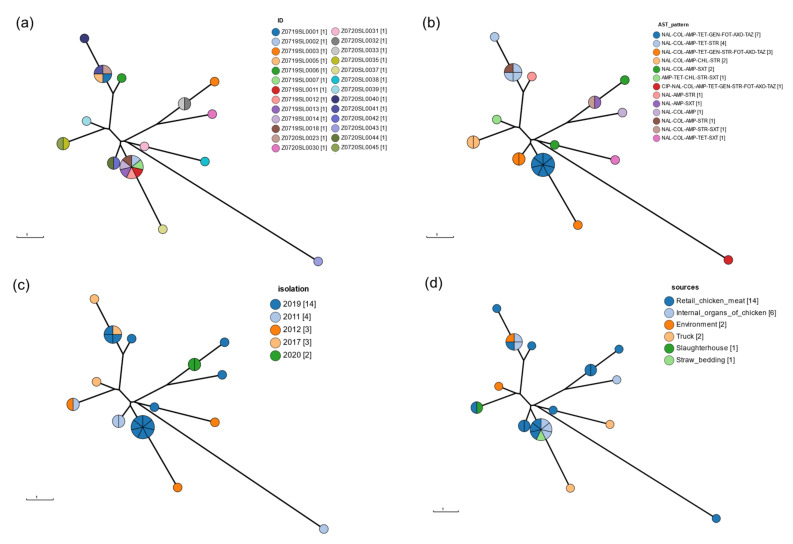
Minimum spanning tree of CgMLST of MDR *Salmonella* Enteritidis. The color of the node indicates (**a**) isolates, (**b**) year of isolation, (**c**) source of isolation, and (**d**) antibiotic resistance profiles.

The results of CgMLST analysis showed that the MDR *Salmonella* Enteritidis strains isolated from different sources and years were clustered together (Figure 1). MDR *Salmonella* Enteritidis strains isolated in 2019 with antibiotic resistance patterns NAL-COL-AMP-TET-GEN-FOT-AXO-TAX clustered with *Salmonella* Enteritidis strains isolated in 2011 and 2012 with antibiotic resistance patterns NAL-COL-AMP-TET-GEN-STR-FOT-AXO-TAX. These strains were isolated from retail chicken meat, internal organs of chicken, trucks, and straw for bedding.

### 3.6. wgSNP Phylogenetic Analysis of the Salmonella Enteritidis Strains

**Figure 2:**
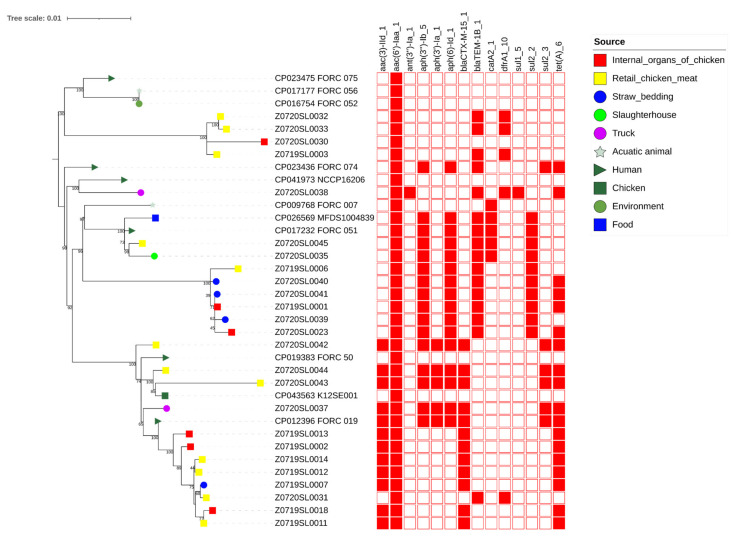
wgSNPs phylogenetic tree of MDR *Salmonella* Enteritidis. Phylogeny was rooted at the midpoint. The presence of antibiotic resistance genes is highlighted in red. The source of isolation of each *Salmonella* Enteritidis is indicated.

The *Salmonella* Enteritidis genomes were clustered into five different groups (I to VII) with one singleton genome (Figure 2). The *Salmonella* Enteritidis strains in the same cluster had similar antibiotic resistance genes. Strains from different sources and different years were grouped with monophyletic clusters. In Cluster I, isolates from humans, the environment, and aquatic animals were clustered together. Four isolates from chicken meat (n = 3) and internal organs of chicken (n = 1) were grouped in Cluster II. Cluster III contained two isolates from truck and human. Cluster IV included four isolates from food, humans, a slaughterhouse, and chicken meat. Six isolates from straw bedding (n = 3), internal organs of chicken (n = 2), and chicken meat (n = 1) were clustered in Cluster V. In Cluster VI, 15 isolates from chicken meat (n = 7), chicken (n = 1), internal organs of chicken (n = 3), humans (n = 2), straw bedding (n = 1), and trucks (n = 1) were included. The *Salmonella* Enteritidis in Clusters II and IV carried *blaTEM-1B* in a plasmid, and those in Cluster V carried *blaTEM-1B* in a chromosome. In Cluster VI, the *Salmonella* Enteritidis strains carrying *blaCTX-M-15* were isolated from chicken meat, internal organs of chicken, straw bedding, humans, and trucks. The Z0720SL0042, Z0720SL0043, and Z0720SL0044 strains were isolated in 2011. The Z0720SL0037 and FORC_019 strains were isolated in 2012 and 2015, respectively. The Z0719SL0002, Z0719SL0007, Z0719SL0011, Z0719SL0012, Z0719SL0013, Z0719SL0014, and Z0719SL0018 strains were isolated in 2019.

## 4. Discussion

To the best of our knowledge, this is the first study that uses WGS to determine the genetic relationship among *Salmonella* Enteritidis strains isolated from humans and poultry sources in Korea. Previous findings obtained by PFGE and MLVA analysis of *Salmonella* Enteritidis strains isolated from humans and poultry sources suggest that *Salmonella* Enteritidis strains have already been transmitted from poultry sources to humans in Korea [14,15]. In this study, results of wgSNP analysis between *Salmonella* Enteritidis strains isolated from humans and poultry sources were consistent with those of the previous PFGE and MLVA studies. The wgSNP phylogenetic analysis revealed a monophyletic relationship, with the support of 100 bootstrap replicates between *Salmonella* Enteritidis isolated from different sources. The *Salmonella* Enteritidis strains in the same cluster had identical antimicrobial resistance gene patterns, indicating that vertical clonal expansion occurred rather than horizontal transmission of the antimicrobial resistance gene.

*Salmonella* is the most common zoonotic foodborne pathogen responsible for gastroenteritis in humans [1]. The rapid emergence of antibiotic resistance in *Salmonella* has been a serious public health problem worldwide [42]. The isolation of MDR *Salmonella*, which is resistant to not only traditional first-line antibiotics such as ampicillin, chloramphenicol, and trimethoprim but also to currently recommended antibiotics, including fluoroquinolones and extended-spectrum cephalosporins, has recently dramatically increased [8,9]. Genotypic and phenotypic antibiotic resistance of the *Salmonella* observed in this study was consistent with the previous findings that MDR *Salmonella* Enteritidis strains isolated showed a high resistance rate to antibiotics commonly used in the Korean poultry industry [5]. Because of the risk of human transmission, the high prevalence of MDR *Salmonella* Enteritidis in the Korean poultry food chain is concerning. MDR *Salmonella* isolated from poultry sources has also been reported in other countries [43,44]. Nevertheless, the prevalence of MDR strains reported herein and in a previous study [5], 50.5% and 50.9, respectively, were higher than that in Spain (9.7%) [45] and China (24.3%) [46].

Colistin was recently used as a last-resort therapeutic option for the therapy of intestinal infections in humans [47]. It is critical to monitor resistance to this agent in isolates from food-producing animals worldwide. Colistin, in turn, has been widely used in the food-animal industry in several countries for the purpose of therapeutic, prophylactic, and growth promotion [48,49]. Colistin was included in poultry formula feeds in Korea prior to 2009. Resistance to colistin can be conferred by various mechanisms, including chromosomal mutation and transmissible genetic mobile elements carrying the colistin resistance gene [50]. Lipid A modification, mediated by mutations in the *pmrHFIJKLM* operon, have been shown to confer resistance to colistin in Enterobacteriaceae [51]. Mutations in *pmrAB* and *pmrLM*, as well as the *AcrAB* efflux pump, have been shown to confer resistance in *S.* Typhymurium [52]. The MDR isolates in this study had no mutations in any of these genes. *mcr* in the plasmid confers resistance by reducing the anionic changes of lipid A, resulting in a lower binding affinity to colistin [51]. To date, 10 variants of *mcr* have been described [53]. The MDR isolates in this study had no *mcr* gene. The absence of colistin resistance, conferring mutations and plasmid-mediated colistin resistance genes in *Salmonella* Enteritidis, has been previously reported [54], suggesting the presence of a novel mechanism for colistin resistance.

Extended-spectrum cephalosporins (ESCs) are the first-line antibiotics for treating salmonellosis and other bacterial infections [55]. *Salmonella* isolates resistant to ciprofloxacin and ESC have increased in recent years [56,57]. Herein, 12 isolates, which were resistant to three third-generation cephalosporins, were clustered together in Cluster VII. Those isolates showed similar antibiotic resistance patterns (NAL-COL-AMP-TET-GEN-FOT-AXO-TAZ). Isolates collected before 2015 showed additional resistance to STR, and one isolate from 2011 showed resistance to both STR and CIP. Most of the antibiotic-resistant genes in strains within Cluster VII were located in the plasmid. MDR *Salmonella* Enteritidis strains in this clade were isolated from various sources and different years. This is a serious public health problem because the vertical clonal expansion of those strains has occurred and been transferred to humans through contaminated food sources.

In conclusion, this study reveals the high prevalence of MDR *Salmonella* in poultry sources in Korea. Considering the location of the antibiotic resistance genes (mainly in the plasmid), analysis of how these plasmids evolve is still warranted to further elucidate the epidemiological emergence of MDR *Salmonella* spp.

## Figures and Tables

**Table 1:** Antibiotic susceptibility profiles of MDR *Salmonella* spp.

Strain	CIP	NAL	IMI	COL	AMP	TET	CHL	AZI	GEN	STR	AMI	SXT	FOT	AXO	FOX	TAZ	Number of Resistance	Number of Antibiotics Classes	Isolation Year	Source of Isolation
Z0719SL0002	I	R	S	R	R	R	S	S	R	S	S	S	R	R	S	R	8	6	2019	Internal organs of chicken
Z0719SL0007	I	R	S	R	R	R	S	S	R	S	S	S	R	R	S	R	8	6	2019	Straw bedding samples
Z0719SL0011	I	R	S	R	R	R	S	S	R	S	S	S	R	R	S	R	8	6	2019	Retail chicken meat
Z0719SL0012	I	R	S	R	R	R	S	S	R	S	S	S	R	R	S	R	8	6	2019	Retail chicken meat
Z0719SL0013	I	R	S	R	R	R	S	S	R	S	S	S	R	R	S	R	8	6	2019	Internal organs of chicken
Z0719SL0014	I	R	S	R	R	R	S	S	R	S	S	S	R	R	S	R	8	6	2019	Retail chicken meat
Z0719SL0018	I	R	S	R	R	R	S	S	R	S	S	S	R	R	S	R	8	6	2019	Internal organs of chicken
Z0720SL0031	I	R	S	R	R	R	S	S	R	S	S	S	R	R	S	R	8	6	2019	Retail chicken meat
Z0719SL0004	I	R	S	S	R	S	S	S	S	S	S	S	R	R	R	R	6	3	2019	Retail chicken meat
Z0719SL0001	I	R	S	R	R	R	S	S	S	R	S	S	S	S	S	S	5	5	2019	Internal organs of chicken
Z0720SL0023	I	R	S	R	R	R	S	S	S	R	S	S	S	S	S	S	5	5	2019	Internal organs of chicken
Z0719SL0008	R	R	S	S	R	S	R	S	S	S	S	S	S	S	R	S	5	4	2019	Straw bedding samples
Z0719SL0009	I	R	S	S	R	R	R	S	S	S	S	R	S	S	S	S	5	5	2019	Slaughterhouse
Z0719SL0010	I	R	S	S	R	R	R	S	S	S	S	R	S	S	S	S	5	5	2019	Slaughterhouse
Z0719SL0021	I	R	S	S	R	R	R	S	S	S	S	R	S	S	S	S	5	5	2019	Slaughterhouse
Z0720SL0026	I	R	S	S	R	R	R	S	S	S	S	R	S	S	S	S	5	5	2019	Slaughterhouse
Z0719SL0003	I	R	S	R	R	S	S	S	S	S	S	R	S	S	S	S	4	4	2019	Retail chicken meat
Z0719SL0005	I	R	S	R	R	S	S	S	S	R	S	S	S	S	S	S	4	4	2019	Retail chicken meat
Z0719SL0017	R	S	S	S	R	R	I	S	S	R	S	S	S	S	S	S	4	4	2019	Slaughterhouse
Z0719SL0022	R	S	S	S	R	R	I	S	S	R	S	S	S	S	S	S	4	4	2019	Slaughterhouse
Z0720SL0029	I	R	S	S	R	S	S	S	S	S	S	S	R	R	I	I	4	3	2019	Retail chicken meat
Z0719SL0006	I	R	S	S	R	S	S	S	S	R	S	S	S	S	S	S	3	3	2019	Retail chicken meat
Z0719SL0015	I	S	S	S	R	R	S	S	S	R	S	S	S	S	S	S	3	3	2019	Slaughterhouse
Z0719SL0016	I	S	S	S	R	R	S	S	S	R	S	S	S	S	S	S	3	3	2019	Slaughterhouse
Z0719SL0019	I	S	S	S	R	R	S	S	S	R	S	S	S	S	S	S	3	3	2019	Internal organs of chicken
Z0719SL0020	I	S	S	S	R	R	S	S	S	R	S	S	S	S	S	S	3	3	2019	Slaughterhouse
Z0720SL0025	I	S	S	S	R	R	S	S	S	R	S	S	S	S	S	S	3	3	2019	Slaughterhouse
Z0720SL0027	I	S	S	S	R	R	S	S	S	R	S	S	I	S	S	S	3	3	2019	Slaughterhouse
Z0720SL0030	I	R	S	R	R	S	S	S	S	S	S	S	S	S	S	S	3	3	2019	Internal organs of chicken
Z0720SL0028	I	S	S	S	R	R	S	S	S	R	S	S	S	S	S	S	3	3	2019	Slaughterhouse
Z0720SL0033	I	R	S	R	R	S	S	S	S	R	S	R	S	S	S	S	5	5	2020	Retail chicken meat
Z0720SL0034	R	R	S	S	R	R	I	S	S	R	S	S	S	S	S	S	5	4	2020	Retail chicken meat
Z0720SL0032	I	R	S	S	R	S	S	S	S	S	S	R	S	S	S	S	3	3	2020	Retail chicken meat
Z0720SL0035	I	R	S	R	R	R	S	S	S	R	S	S	S	S	S	S	5	5	2012	Slaughterhouse
Z0720SL0037	I	R	S	R	R	R	S	S	R	R	S	S	R	R	S	R	9	6	2012	Truck
Z0720SL0038	S	S	S	S	R	R	R	S	S	R	S	R	S	S	S	S	5	5	2012	Truck
Z0720SL0039	I	R	S	R	R	R	S	S	S	S	S	R	S	S	S	S	5	5	2017	Environment
Z0720SL0040	I	R	S	R	R	R	S	S	S	R	S	S	S	S	S	S	5	5	2017	Retail chicken meat
Z0720SL0041	I	R	S	R	R	S	R	S	S	R	S	S	S	S	S	S	5	5	2017	Environment
Z0720SL0042	I	R	S	R	R	R	S	S	R	R	S	S	R	R	S	R	9	6	2011	Retail chicken meat
Z0720SL0043	R	R	S	R	R	R	S	S	R	R	S	S	R	R	S	R	10	6	2011	Retail chicken meat
Z0720SL0044	I	R	S	R	R	R	S	S	R	R	S	S	R	R	S	R	9	6	2011	Retail chicken meat
Z0720SL0045	I	R	S	R	R	S	R	S	S	R	S	S	S	S	S	S	5	5	2011	Retail chicken meat

Ciprofloxacin (CIP), nalidixic acid (NAL), imipenem (IMI), colistin (COL), ampicillin (AMP), tetracycline (TET), chloramphenicol (CHL), azithromycin (AZI), gentamicin (GEN), streptomycin (STR), amikacin (AMI), trimethoprim/sulfamethoxazole (SXT), cefotaxime (FOT), ceftriaxone (AXO), cefoxitin (FOX), ceftazidime (TAZ); R, resistant; S, susceptible; I, intermediate.

**Table 2:** The genomic features of the MDR *Salmonella* spp.

Sample Name	Data Output (gb)	Fold Coverage (X)	Chromosome Size (bp)	Number of Plasmid	Serovar
Z0720SL0023	1.8	375.0	4,783,705	1	Enteritidis
Z0719SL0001	5.2	1083.3	4,783,876	1	Enteritidis
Z0719SL0004	1.8	375.0	4,673,348	0	Virchow
Z0719SL0003	6.4	1333.3	4,679,604	3	Enteritidis
Z0719SL0002	5.9	1229.2	4,680,702	1	Enteritidis
Z0719SL0005	1.75	364.6	4,779,036	1	Enteritidis
Z0719SL0006	5.9	1229.2	4,779,850	0	Enteritidis
Z0719SL0007	2.8	583.3	4,681,486	1	Enteritidis
Z0719SL0008	2.5	520.8	4,670,331	2	Virchow
Z0719SL0011	4.5	937.5	4,681,460	1	Enteritidis
Z0719SL0012	4.7	979.2	4,681,475	1	Enteritidis
Z0719SL0009	2.1	437.5	4,809,470	1	Albany
Z0719SL0010	2.1	437.5	4,809,485	2	Albany
Z0719SL0013	4.8	1000.0	4,683,147	1	Enteritidis
Z0719SL0014	5.3	1104.2	4,678,918	1	Enteritidis
Z0719SL0018	1	208.3	4,681,459	1	Enteritidis
Z0719SL0019	7	1458.3	4,843,579	1	Agona
Z0720SL0025	0.62	129.2	4,878,409	1	Agona
Z0719SL0021	2.85	593.8	4,844,531	0	Albany
Z0720SL0026	0.42	87.5	4,844,485	0	Albany
Z0720SL0027	0.6	125.0	4,593,080	1	Virchow
Z0720SL0028	2.09	435.4	4,677,146	2	Virchow
Z0719SL0015	2.7	562.5	4,843,592	1	Agona
Z0719SL0016	5	1041.7	4,843,581	1	Agona
Z0719SL0017	4.9	1020.8	4,877,928	1	Agona
Z0719SL0022	2.05	427.1	4,877,150	1	Agona
Z0719SL0020	2.05	427.1	4,878,418	1	Agona
Z0720SL0030	0.51	106.3	4,547,043	0	Enteritidis
Z0720SL0031	0.54	112.5	4,681,359	4	Enteritidis
Z0720SL0032	3.3	687.5	4,679,600	4	Enteritidis
Z0720SL0033	1.05	218.8	4,679,611	4	Enteritidis
Z0720SL0034	1.1	229.2	4,670,318	0	Virchow
Z0720SL0035	1.31	272.9	4,680,380	1	Enteritidis
Z0720SL0037	1.55	322.9	4,680,192	3	Enteritidis
Z0720SL0038	1.56	325.0	4,680,091	2	Enteritidis
Z0720SL0039	1.53	318.8	4,807,544	2	Enteritidis
Z0720SL0040	2.55	531.3	4,782,444	1	Enteritidis
Z0720SL0041	1.32	275.0	4,783,583	1	Enteritidis
Z0720SL0042	1.29	268.8	4,678,693	1	Enteritidis
Z0720SL0043	1.47	306.3	4,664,874	1	Enteritidis
Z0720SL0044	1.36	283.3	4,680,669	1	Enteritidis
Z0720SL0045	1.49	310.4	4,679,466	1	Enteritidis

**Table 3:** Antibiotic resistance patterns and antibiotic resistance genes in MDR *Salmonella* Enteritidis strains.

Antibiotic Resistance	Sources of Isolation	Antibiotic Resistance Gene	No. of Isolates	No. of Antibiotics	No. of Classes
NAL-COL-AMP-TET-GEN-FOT-AXO-TAZ	Retail chicken meat	aac(6′)-Iaa_1 aac(3)-IId_1 blaCTX-M-15_1 tet(A)_6	3	8	6
	Straw for bedding		1		
	Internal organs of chicken		3		
NAL-COL-AMP-TET-GEN-STR-FOT-AXO-TAZ	Retail chicken meat	aac(6′)-Iaa_1 sul2_3 aph(3″)-Ib_5 aph(6)-Id_1 aph(3′)-Ia_1 aac(3)-IId_1 blaCTX-M-15_1 tet(A)_6	2	9	6
	Truck		1		
CIP-NAL-COL-AMP-TET-GEN-STR-FOT-AXO-TAZ	Retail chicken meat	aac(6′)-Iaa_1 aac(3)-IId_1 sul2_3 aph(3″)-Ib_5 aph(6)-Id_1 aph(3′)-Ia_1 tet(A)_6 blaCTX-M-15_1	1	10	6
NAL-COL-AMP-TET-STR	Retail chicken meat	aac(6′)-Iaa_1 blaTEM-1B_1 aph(6)-Id_1 aph(3″)-Ib_5 tet(A)_6 aph(6)-Id_1 aph(3″)-Ib_5 sul2_2	1	5	5
	Environment		1		
	Internal organs of chicken		2		
NAL-COL-AMP-STR-SXT	Retail chicken meat	aac(6′)-Iaa_1 blaTEM-1B_1 dfrA1_10	1	5	5
NAL-COL-AMP-CHL-STR	Slaughterhouse	aac(6′)-Iaa_1 blaTEM-1B_1 aph(6)-Id_1 aph(3′)-Ib_5 tet(A)_6 aph(6)-Id_1 aph(3″)-Ib_5 sul2_2	1	5	5
	Retail chicken meat		1		
NAL-COL-AMP-TET-SXT	Truck	aac(6′)-Iaa_1 blaTEM-1B_1 aph(6)-Id_1 aph(3″)-Ib_5 tet(A)_6 aph(6)-Id_1 aph(3″)-Ib_5 sul2_2	1	5	5
AMP-TET-CHL-STR-SXT	Environment	aac(6′)-Iaa_1 catA2_1 sul2_2 aph(3″)-Ib_5 aph(6)-Id_1 blaTEM-1B_1	1	5	5
NAL-COL-AMP-SXT	Retail chicken meat	aac(6′)-Iaa_1 blaTEM-1B_1 dfrA1_10	2	4	4
NAL-COL-AMP-STR	Retail chicken meat	sul2_2 aph(3″)-Ib_5 aph(6)-Id_1 blaTEM-1B_1 aac(6′)-Iaa_1	1	4	4
NAL-AMP-STR	Retail chicken meat	sul2_2 aph(3″)-Ib_5 aph(6)-Id_1 blaTEM-1B_1 aac(6′)-Iaa_1	1	3	3
NAL-COL-AMP	Internal organs of chicken	aac(6′)-Iaa_1	1	3	3
NAL-AMP-SXT	Retail chicken meat	dfrA1_10 blaTEM-1B_1 aac(6′)-Iaa_1	1	3	3

## Data Availability

The complete genomes of *Salmonella* spp. in this study are available from the GenBank under BioProjects PRJNA658425 and PRJNA780385.
